# The Role of Grafting in the Resistance of Tomato to Viruses

**DOI:** 10.3390/plants9081042

**Published:** 2020-08-16

**Authors:** Roberta Spanò, Massimo Ferrara, Donato Gallitelli, Tiziana Mascia

**Affiliations:** 1 Department of Soil, Plant and Food Sciences, University of Bari “Aldo Moro”, 70126 Bari, Italy; donato.gallitelli@uniba.it (D.G.); tiziana.mascia@uniba.it (T.M.); 2 Institute of Sciences of Food Production (ISPA)—CNR, 70126 Bari, Italy; massimo.ferrara@ispa.cnr.it

**Keywords:** vegetable grafting, RNAi, wound and pathogen response, plant viruses, disease tolerance/resistance, tomato ecotype

## Abstract

Grafting is routinely implemented in modern agriculture to manage soilborne pathogens such as fungi, oomycetes, bacteria, and viruses of solanaceous crops in a sustainable and environmentally friendly approach. Some rootstock/scion combinations use specific genetic resistance mechanisms to impact also some foliar and airborne pathogens, including arthropod or contact-transmitted viruses. These approaches resulted in poor efficiency in the management of plant viruses with superior virulence such as the strains of tomato spotted wilt virus breaking the Sw5 resistance, strains of cucumber mosaic virus carrying necrogenic satellite RNAs, and necrogenic strains of potato virus Y. Three different studies from our lab documented that suitable levels of resistance/tolerance can be obtained by grafting commercial tomato varieties onto the tomato ecotype Manduria (Ma) rescued in the framework of an Apulian (southern Italy) regional program on biodiversity. Here we review the main approaches, methods, and results of the three case studies and propose some mechanisms leading to the tolerance/resistance observed in susceptible tomato varieties grafted onto Ma as well as in self-grafted plants. The proposed mechanisms include virus movement in plants, RNA interference, genes involved in graft wound response, resilience, and tolerance to virus infection.

## 1. Introduction

The tomato virome consists of at least 136 characterized virus species threatening tomato production worldwide plus a number of new viruses that are being progressively identified by next-generation sequencing [1]. The role of these not yet characterized viruses in pathogenesis, disease development, and crop losses is unknown. Characterized and economically relevant viruses infecting tomato include the strains of tomato spotted wilt virus (TSWV) breaking the resistance conferred by the SW5 gene (Sw5-RB) (TSWV), strains of cucumber mosaic virus (CMV) harboring harmful variants of their satellite RNAs (satRNA), and isolates of potato virus Y (PVY) necrogenic to tomato, which appeared since the 1980s, and quickly became one of the most important tomato diseases in the Mediterranean countries. TSWV, CMV, and PVY are transmitted by arthropods and are included among the top ten economically important plant viruses [2] for which efficient and environmentally friendly methods of control are not yet available. Means of natural transmission, extended host range, and appearance of new or recombinant strains with increased virulence may account for control failures. CMV infects approximately 1300 monocots and dicots within 500 genera of more than 100 botanical families [3] including crop and wild species, which are important for virus persistence in open field [2,4]. TSWV host range is also large, consisting of at least 1090 species among crop and wild plants in 84 botanical families [2,5,6]. Finally, PVY has a relatively narrower host range compared to TSWV and CMV but, similar to them, new and emerging strains may cause severe epidemics in pepper, potato, tobacco, and tomato [2,7,8]. In spite of several attempts to obtain suitable levels of resistance by classical breeding or transgenesis [4,8,9,10,11], field control of these viruses is still based on routine pesticide sprays against vectors, which proved scantily efficient because of the stylet-borne nature of the non-persistent transmission of CMV and PVY mediated by aphids and the complex persistent propagative relationship between thrips and TSWV, which replicates in both plants and thrips, thus leading to the continuous emergence of resistance-breaking strains and new species [12]. On the other hand, introgression of potential resistance genes into commercial crops proved ineffective against virus strains with high genome plasticity and superior virulence. Several recessive (sw2, sw3, and sw4) and dominant (Sw1a and Sw1b) resistance genes against TSWV have been reported and incorporated into commercial tomato cultivars (see for review [9] and references quoted therein) but their resistance was isolate-specific and, therefore, it was quickly overcome by TSWV infection. Only the Sw-5 gene cluster originated from *Solanum peruvianum* provided durable and stable resistance against TSWV isolates from different geographic areas. However, large deployment of such Sw5-TSWV-resistant tomato varieties has resulted in the onset of RB isolates overcoming the resistance by single-amino acid substitutions in the TSWV NSM protein [13,14]. Resistance sources against CMV have been identified within the genus *Solanum*, but all were strain specific and/or quantitative [15]. Thus, CMV-resistant tomato varieties are not yet commercially available. Transgenic resistance of tomato varieties expressing the CMV coat protein or beneficial satRNAs has been shown to be partially [16,17] or highly efficient [18,19], respectively, in field trials. However, implementation of satRNA-mediated protection in agriculture is still a matter of debate for potential risks [20]. Necrogenic strains of PVY induce necrotic lesions on tomato leaves followed by a streak on stems with all the commercial hybrids and varieties commonly grown in greenhouses and open field. Therefore, unlike what is described in potato, pepper, and tobacco, PVY did not reveal any strain specialization to infect tomato [21]. This, in theory, should have facilitated the search for PVY resistance genes to be introgressed in tomato. According to Parrella et al. [22], resistance against PVY and another potyvirus tobacco etch virus has been identified in the wild tomato accession “PI 247087.” The resistance gene corresponds to a eukaryotic translation initiation factor that interacts with viral VPg and prevents virus accumulation in inoculated tissues [23]. In spite of these results, PVY-resistant tomato varieties are not yet available on the market. Thus, introgression of potential resistance genes into commercial crops proved ineffective against virus strains with high genome plasticity and superior virulence.

Therefore, alternative integrated management strategies were exploited.

Suitable levels of resistance to abiotic and biotic stresses have been successfully obtained in vegetable annual crops by grafting two genotypes selected as scion and rootstock. Actually, grafting offers a rapid surgical alternative to the time-consuming classical breeding or transgenesis to combine desired characteristics of rootstock and scion in vegetable crops [24,25].

Ideally, scion must cope with crop performance and yield required by farmers and fruit quality and nutritional value required by consumers [26]. The rootstock should guarantee cultivation under adverse environments such as salinity, nutrient deficiency, drought, pollutants, and soil-borne pathogens such as fungi, oomycetes, bacteria, and viruses [27,28,29,30,31,32,33]. Among vegetable crops, tomato, eggplant, sweet pepper, watermelon, melon, and cucumber are commonly and economically grafted in Asia, Europe, and North America. To give some examples (reviwed by [34]), watermelon, melon, and cucumber are prevalently grafted onto *Cucurbita maxima* × *C. moschata* rootstocks such as “Shintoza,” “Ps1313,” “TZ 148” or onto *C. moschata* “Jinxinzhen,” *C. pepo* “Brava,” and *C. ficifolia* in the case of cucumber. Tomato has been grafted mainly onto tomato genotypes and interspecific hybrids such as the *Solanum lycopersicum* × *S. habrochaites* “Maxifort” and “Beaufort” rootstocks, which improve crop yield, tolerance to soilborne pathogens and fruit quality, favoring higher accumulation of phenolic compounds, vitamin C, lycopene, and flavonoids in fruits of grafted plants [35]. Eggplant has been grafted traditionally on *Solanum* spp. wild species such as *S. integrifolium*, *S. torvum*, and *S. sisymbriifolium*, which provide high level of resistance to bacterial wilt, Fusarium wilt, Verticillium wilt, and root-knot nematodes. Compared to tomato and eggplant, grafted sweet peppers are not typically commercialized. Fruit shape is important in pepper crops but certain rootstocks can modulate gene expression and fruit development of the scion. Tsaballa et al. [36] demonstrated that phenotypic change in sweet pepper can be inherited for up to two generations of seed produced by these progenies. Nonetheless, suitable levels of resistance against root-knot nematodes and *Phytophthora capsici* were attained in sweet pepper grafted on *Capsicum annuum* accessions “AR96023” and “AF2638,” respectively. Finally, this list could not be exhaustive without including the control of Verticillium wilts obtained by grafting seed-propagated globe artichoke hybrids onto cardoon [37] as well as the list of potential rootstocks with special characteristics to manage biotic and abiotic stresses in tomato, eggplant, chili, potato, cucumber, muskmelon, pumpkin, and wax gourd reported by Kumar et al. [38].

Grafted tomato plants were implemented in the early 1960s but their use aroused little interest because farmers were particularly discouraged by the higher price of grafted plants compared to non-grafted seedlings. Recent surveys report that the price of grafted tomato seedlings for fresh market tomato production is between 0.4 to 1.2 USD per grafted seedling in the USA and some Asian countries, including Japan and Korea, and between 0.6 to 1.2 EUR per grafted seedling in Spain and some European countries [39]. Nonetheless, actual estimates suggest between 20% and 40% of tomatoes are grafted [39] and their implementation is rapidly increasing all over the world in organic and environmentally friendly crops for the control of soil-borne pathogens as an alternative to the banned methyl bromide and to mitigate effects of abiotic stresses.

The higher cost of the grafted plantlets is now compensated by a number of advantages, which include production increase and earliness of the harvest with a reduced number of plants, rational use of irrigation water, use of fewer fertilizers, and elongation of the crop cycle. A good rootstock/scion combination usually guarantees a robust root system and the maintenance of good vegetative vigor and resistance to deal with abiotic and biotic stresses until the end of the farming cycle [27]. All these aspects guarantee net returns large enough to make grafted tomatoes significantly more profitable than non-grafted seedlings [40]. Grafting is a viable approach also to limit damage by viruses transmitted through soil such as melon necrotic spot virus (MNSV) vectored by *Olpidium* sp. or the contact-transmissible tobacco mosaic virus (TMV), which reaches the soil in infected plant debris or as seed-coat contaminant. Rootstocks with specific characteristics have been developed and deployed for the control of MNSV in muskmelon and watermelon [41,42,43] and of TMV in tomato [38]. However, the majority of plant viruses is airborne by arthropods and pollen or transmitted by contact or aerosol [44,45] (Table 1).

Some promising attempts were recently made to reduce incidence of tomato yellow leaf curl virus, TSWV, and pepino mosaic virus in different scion-rootstocks combinations [46,47,48,49].

## 2. Why Grafting?

Samuel [50] described a general pattern of translocation of tobacco mosaic virus (TMV) into plant tissues suggesting that after entering tobacco leaf tissue, TMV particles primarily reach roots through phloem sieve tubes and between 2 to 3 days post-inoculation (dpi) they follow phloem sap in the reverse way up, bypass the old leaves, and invade systemically the young plant tissues [51]. Andrianifahanana et al. [52] reported a similar pattern of virus translocation in pepper of the potyvirus pepper mottle virus. The authors documented virus movement from the inoculated leaf down the stem toward the roots via the external phloem. Then the virus entered internal phloem at the cotyledon node and rapidly spread upward to the young tissues. A phloem-translocation pattern has been described also for cauliflower mosaic virus [53], cherry leafroll virus [54], and TMV [55] (reviewed in [56,57,58]). Internal and external phloem is present in the stem of *Solanaceae* and of some other plant families such as *Cucurbitaceae* and corresponds to the adaxial and abaxial phloem strands of leaf veins and petioles. In tomato leaflets the xylem is in the center of the vein with the phloem distributed on both the adaxial and abaxial sides of the bundle. The abaxial phloem exports sugars from the leaf in a descending direction to roots. Thus, a virus entering abaxial leaf phloem is expected to follow transport of photoassimilates down to roots, according to the source-sink relationship [59]. For potyviruses, virus loading in the external phloem depends on the virus-host combination [56] and for potato virus A it is controlled by the virus-coded Vpg [60]. On the contrary, a complex structure of CMV containing viral RNA, coat protein, and the 3a movement protein loads into sieve elements of minor veins of *Nicotiana clevelandii*. The complex successively assembles into complete virus particles within minor vein sieve elements [58,61]. Unfortunately no such data are available for TSWV.

Results from these studies were seminal to our idea to exploit grafting as a strategy to control airborne viral infections in tomato.

This review gives a brief account of the results and proposes mechanisms probably involved in the resistance/tolerance observed in grafted plants of the three cases-study carried out in our laboratory to manage one Sw5-RB strain of TSWV [62], two CMV strains supporting stunting and necrogenic satRNAs [63], and one recombinant PVY strain necrogenic to tomato [64].

## 3. Screening of *Solanum* spp. Germplasm

Local tomato ecotypes and commercial hybrids and *Solanum* spp. genotypes were screened for resistance/tolerance to challenge inoculation of specific strains of TSWV, CMV, and PVY characterized by superior virulence.

Screening for potential rootstocks privileged local tomato ecotypes found and characterized in the framework of regional actions aimed at preserving biodiversity of vegetable crops in Apulia (southern Italy). Selected ecotypes were Fiaschetto (Fi), Giallo invernale (Gi), Manduria (Ma), Morciano (Mor), Racalino (R), and Regina (Re). Screenings included also *Solanum integrifolium*, *S. nigrum*, *S. torvum*, eggplant cv. Molfettese (Mo), and commercial tomato hybrids, Faino (Fa), Messapico (Me), Taylor (Ta) or not Pullrex (Pu), carrying the Sw5-resistance gene to TSWV and the tomato variety UC82 (UC) as highly susceptible control. Tests were carried out with cleft-grafted and non-grafted plants rub-inoculated on the first leaf above the graft junction with sap extracted from infected plants and grown under glasshouse [62,63,64] or exposed to field inoculum (this review). According to Kumar et al. [38], complete repair of graft wound would be achieved between 7 and 10 days after grafting, thus we were confident that by the time of inoculation, continuous cambial connections between scion and rootstock had been restored. In glasshouse tests, plants were grown and maintained at 24 ± 2 °C with 16 h photoperiod and monitored daily for disease symptoms. Accumulation of viral RNA was estimated by dot blot or tissue print hybridization in samples collected from inoculated and mock-inoculated plants at 14, 21, and 28 days post-inoculation (dpi). Plant tissues were ground in the presence of an alkaline solution and spotted onto positively charged nylon membranes that were hybridized overnight with virus-specific DIG-labeled RNA or DNA probes. Chemiluminescent hybridization signals were used to detect and quantify the accumulation of viral RNA by using *Glyceraldehyde 3-phosphate dehydrogenase* (*GAPDH*) as housekeeping gene for normalization [65]. Viral genomic RNAs and virus-specific small interfering RNAs were separated on agarose or polyacrylamide gels, respectively, and detected by northern blot hybridization with specific radiolabeled or DIG-labeled probes [62,63] or by NGS sequencing [64].

For field tests, three biological replicates of each plant at first branching stage were transplanted according to a fully randomized block scheme. In 2016 and 2017, two experimental fields were set up between March and April using 270 and 180 plants per field, respectively. Data were collected before the middle of August, at the end of vegetative season.

### 3.1. Results with TSWV

*S. nigrum* and UC showed systemic symptoms ranging from severe mosaic, to leaf and stem necrosis and plant death in response to infection of the Sw5-RB strain TSWV-CiPz, whereas Ma, Mo, and *S. integrifolium* showed mild mosaic and, more interestingly, recovered from viral disease symptoms between 21 and 28 dpi (Table 2). The lowest levels of viral RNA were detected in Ma, Mo, and *S. integrifolium*, therefore these genotypes were selected as rootstocks to prepare graft combinations. Self-grafted UC/UC, Ma/Ma, Fa/Fa, Me/Me, and the Me and Fa scions grafted onto Ma showed mild mosaic. All these plants recovered from disease symptoms and displayed increased growth, abundant leaf canopy, and root development, compared to non-grafted infected plants. Oneweek later, also UC/Mo, and Pu/Pu recovered from disease symptoms. Recovery was not observed with non-grafted UC and Pu. Viral RNA load was markedly reduced in the Me/Ma graft combination as well as in all scions of the self-grafted combinations (Table 2).

**Figure 1:**
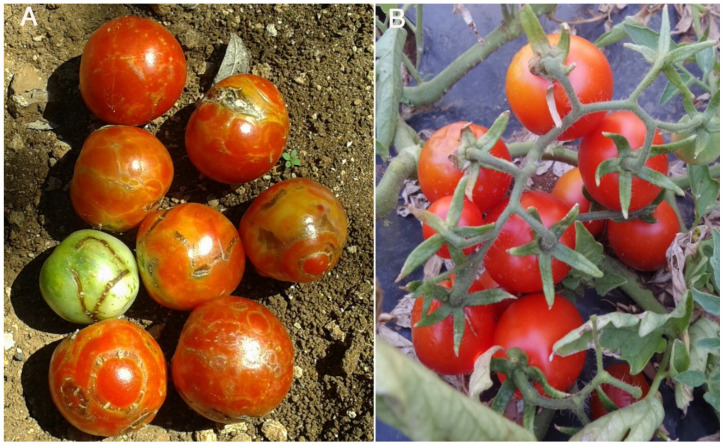
Severe symptoms of a Sw5-RB strain of TSWV on tomato cv Regina (**A**) compared to the same variety grafted onto Manduria (**B**) during 2016 field tests.

In a field test set up in 2016, the only virus detected was a Sw5-RB TSWV strain identified by targeting NS_m_ sequences flanking the *Mae*I restriction site with RT-PCR RFLP [66] and rub-inoculation onto the commercial tomato hybrids Diaz and York carrying the Sw5 resistance gene [67]. Field infection damaged 7–8% of the plants with necrotic symptoms on leaves and fruits. Among the tomato ecotypes evaluated, Regina (Re) proved the most susceptible (Figure 1) but despite the overall low incidence of viral infection in the field plants self-grafted or grafted onto Ma showed a productive advantage compared to the non-grafted counterparts. In a second field test set up in 2017, we exposed to field inoculum the Sw5 commercial tomato hybrid Taylor self-grafted or grafted onto Ma. Non-grafted Taylor plants served as control. Again, the only virus detected was an Sw5-RB TSWV strain. Graft combinations Taylor/Taylor and Taylor/Ma produced between 6 and 9 kg/plant, whereas the non-grafted Taylor counterparts produced between 4 and 5 kg/plant, which is thought to be the standard production of this hybrid. In addition, the non-grafted Taylor plants, showed sporadic symptoms of Sw5-RB TSWV infection and about ten days delay in fruit ripening, compared to the grafted plants.

### 3.2. Results with CMV

CMV infections in tomato crops became suddenly relevant around the 1980s after the introduction of the so-called “Asian” strains characterized by superior aggressiveness compared to endemic CMV strains [68]. After initial severe outbreaks between 1988 and 1992, CMV has been detected sporadically in tomato fields although outbreaks may still occur after very mild winters, which may lead to huge increases in overwintering aphid populations that spread the virus from infected foci in wild plants communities into the crops [68,69]. Therefore we limited our study to glasshouse tests. Infection of CMV-TTS carrying a tomato top stunting satRNA induced leaf distortion and apical stunting in UC and Ma (Table 3), which persisted in UC until 30 dpi but not in Ma plants that recovered from disease symptoms. Symptoms elicited in non-grafted plants were very similar to those of grafted plants but, by 21 dpi, the latter fully recovered from disease symptoms (Table 3). Viral RNA loads in Ma were about 1.6-fold lower than in UC whereas no significative differences were estimated among grafted and self-grafted plants. Systemic infection of CMV-77 carrying a necrogenic satRNA induced stunting and severe stem and leaf necrosis in both Ma and UC, between 15 and 18 dpi (Table 3). Ma plants transiently recovered from necrosis between 18 and 21 dpi but by 30 dpi, these plants also died (Table 3). Non-grafted genotypes showed a more severe and rapid disease progression than grafted plants. Necrosis developed in all UC self-grafted plants and in one out of the four UC/Ma plants but none of the plants died. Interestingly, self-grafted Ma plants recovered from necrosis by 21 dpi (Table 3). Because of the occurrence of a necrotic phenotype, we monitored the accumulation of viral RNA at 15 dpi. At this time-point, viral RNA load in Ma was 8-fold lower than in UC and this ratio was substantially similar among the three graft combinations tested (Table 3).

### 3.3. Results with PVY

PVY isolates infecting tomato show high genetic diversity, including genetic recombination. One of such recombinants, denoted PVY^C^-to, was characterized as an interlineage recombinant isolate of the PVY^C^ group and associated with necrotic spots and vein necrosis on the leaflets and pale yellow spots scattered on fruit skin of a table tomato variety [70].

In two independent glasshouse experiments, PVY^C^-to infected systemically UC and Ma. UC infection was characterized by mosaic, leaf blade reduction, and twisting with some necrotic spots scattered on the leaf surface. Conversely, infection in Ma was substantially asymptomatic but viral RNA loads at 14 dpi did not differ significantly between UC and Ma, despite the differences in symptoms. Infection of PVY^C^-to in the three graft combinations induced a mild distortion of leaf margin and a slightly reduced growth. Viral RNA loads at 14 dpi did not differ significantly among the three graft combinations but was approximately 2.5-fold higher than in non-grafted plants. Despite the difference in viral RNA loads, non-grafted Ma and all grafted plants recovered from disease symptoms by 21 dpi with a mean of 3-fold reduction in viral RNA loads compared with the estimates at 14 dpi sampling time. Non-grafted UC plants did not recover from disease symptoms; rather, they suffered increased disease severity and a further 2-fold increase in viral RNA loads by passing from 14 to 21 dpi (Table 4).

## 4. Proposed Models

### 4.1. Virus Movement in Grafted Plants

**Figure 2:**
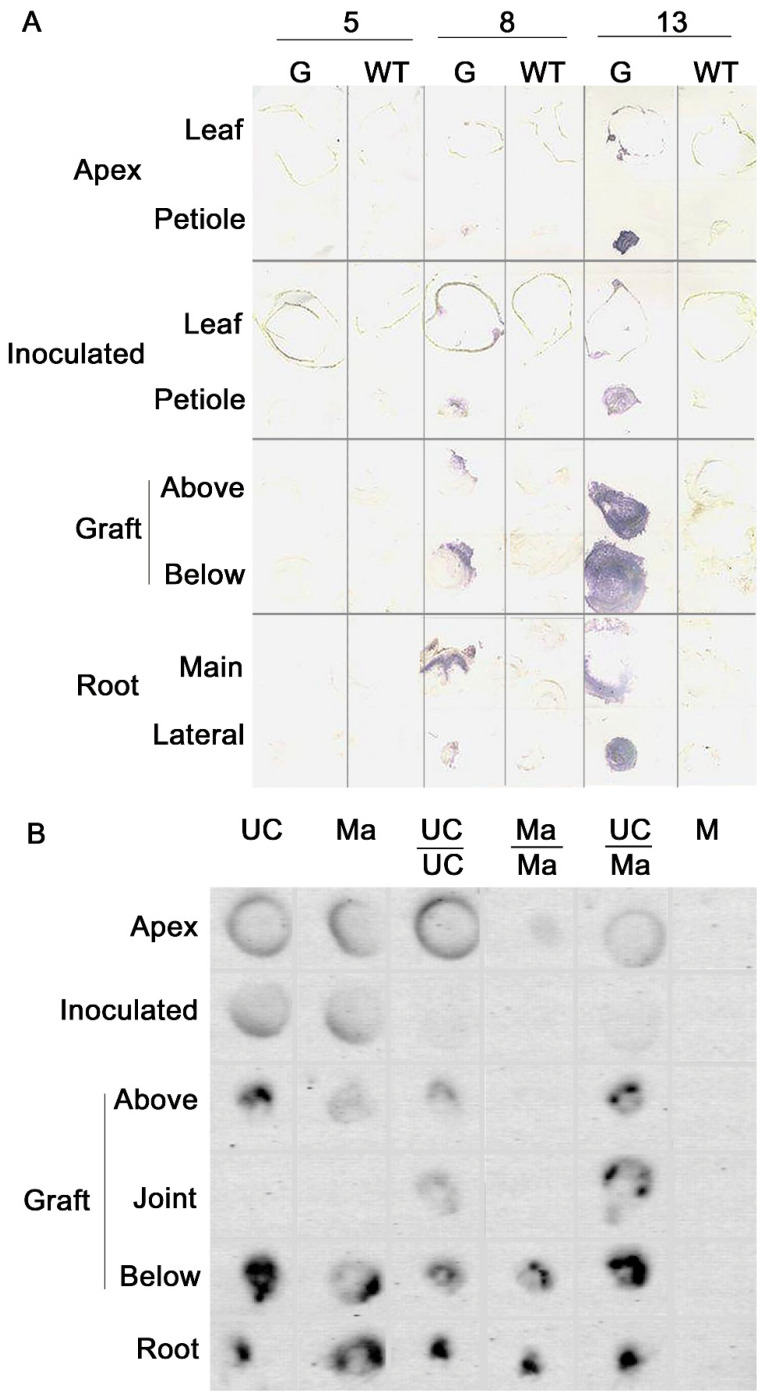
Detection of TSWV in sections of self-grafted (G) and non-grafted (WT) *N. benthamiana* plants at 5, 8, and 13 dpi with TSWV-CiPz. (**A**). Cross sections were prepared from apex, inoculated leaf, above and below graft junction, and from main and lateral roots. Virus was detected by using an antiserum against the NSs protein. (**B**). Detection of TSWV-CiPz RNA in UC, Ma, UC/UC, Ma/Ma, and UC/Ma at 19 dpi. Viral RNA was detected by tissue print hybridization with a DIG-labelled RNA probe for TSWV M RNA. Cross sections were prepared from apex, stem junction of the inoculated leaf, above, at and below the graft junction and from principal root. M = mock-inoculated UC plant.

Given the airborne nature of TSWV, we studied virus movement in grafted plants of *Nicotiana benthamiana* and tomato (Figure 2). At 5 dpi, TSWV-CiPz was detected in the inoculated and in the apical leaf of *N. benthamiana* whereas downward virus movement was evident only at 8 dpi by high virus accumulation above and below the graft union. Increased virus-specific signal at 13 dpi in the petiole of the apical leaf suggested virus movement toward the plant apex. By analogy with potyviruses [52] and TMV [57], the interval of 5 dpi between the inoculation and detection of TSWV-CiPz in the inoculated leaf might mirror initial viral replication and protein expression in rub-inoculated epidermal cells. Cell-to-cell movement then, may have led infection outwards to the immediately adjacent cells, with a relatively slow process estimated in one cell/2 h so that infection boundary advanced only a small distance per day [57]. Leaves on the same side of the stem having phloem traces adjacent to one another and carrying upward plant material and virus [59] may account for the simultaneous detection of TSWV-CiPz in the apex and in the inoculated leaf of *N. benthamiana*. Tissue-print hybridization of UC and Ma plants showed a virus movement pattern similar to that observed in *N. benthamiana*. The virus did not move out from the inoculated leaf until 4 dpi to descend quickly through the petiole down to the graft and roots. Distribution and accumulation of TSWV-CiPz RNA in non-grafted and grafted tomato was analyzed at 19 dpi (Figure 2). Viral RNA was detected in all sections but the hybridization signal was much weaker in the apex and inoculated leaf than in the roots, suggesting a reduced viral RNA load. The reduction was particularly marked in self-grafted Ma plants.

### 4.2. RNA Interference

RNA interference (RNAi) is a defensive reaction against biotic stresses conserved in eukaryotic organisms. The reaction includes the synthesis of invasive double-stranded RNAs (dsRNA), such as the replicative intermediates or secondary structures of single stranded RNA (ssRNA) viruses, which are recognized and diced into 21–24 small RNA (sRNA) duplexes by conserved type III ribonucleases of the Dicer-like (DCL) protein family. The small dsRNA fragments denoted primary viral short interfering RNAs (primary vsiRNAs) are then loaded onto proteins of the Argonaute (AGO) family to build up RNA-induced silencing complexes (RISCs) that are the effectors of the process. RISCs removes one of the two strands from primary vsiRNAs and uses the other to detect and degrade viral sequences complementary to the RISC-retained vsiRNA strand [71,72]. After interference initiation in one or few cells, plant RNA-dependent RNA polymerases (RDR1 or RDR6) increase the amount of dsRNA as a DCL substrate for the production of secondary vsiRNAs and amplification of the RNAi signal. Thus, initial RNAi signal spreads progressively to adjacent cells through plasmodesmata and over long distance through plant phloem [73,74,75]. Interestingly, systemic movement of RNAi signal was demonstrated by grafting and in studies with viruses and viroids [76,77,78,79]. Another point in favor of a tolerance/resistance mechanism mediated by RNAi is the occurrence of a recovery phenotype prevalently in grafted plants and independently from the challenging virus. Recent evidences suggested that recovery from virus-induced disease symptoms might be the consequence of a robust delivery of secondary vsiRNAs produced by the DCL4/RDR6/SGS3 RNAi pathway to saturate the viral suppressor of RNA silencing (VSRs) activity involved in determining disease symptom with the formation of a virus tolerant condition in infected tissues [80]. Therefore, we evaluated whether a differential response based on RNAi could be involved in the different susceptibility to TSWV-CiPz, CMV-TTS and CMV-77 observed among the *Solanum* spp. genotypes tested. Increase in the population of small RNAs (sRNAs) purified at 21 dpi was clearly seen in non-grafted UC and Ma plants, upon TSWV-CiPz infection [62]. Results from hybridization of the three L, M, and S genomic RNAs of TSWV-CiPz with radiolabeled sRNA probes suggested that the majority of sRNAs in the total pool corresponded to vsiRNAs produced from genomic RNA S whereas those corresponding to genomic segments L and M were apparently less represented [62]. The TSWV S RNA encodes the non-structural protein NSs that has VSRs activity [81,82,83]. Quantitative results from hybridization suggested also a stronger accumulation of vsiRNAs in Ma than in UC plants in agreement with the 3-fold reduction of viral RNA and the recovery phenotype elicited by Ma plants compared to UC plants (Table 2). Relative abundance of transcripts of *AGO*1, *AGO*4, *DCL*1, *DCL*2, *DCL*4, *PAZ*, *RDR*1, and *RDR*6 (orthologs of *Arabidopsis thaliana*) in roots and leaves of Ma plants at 21 dpi with TSWV-CiPz suggested that Ma plants could elicit a stronger RNAi response compared to UC even when used as rootstock. For example, *AGO*4 and *RDR*6, were up-regulated in leaves of UC and Me when grafted onto Ma [62]. Thus, the symptom attenuation and reduced accumulation of TSWV-CiPz RNA estimated in UC and Me when grafted onto Ma strengthens the involvement of an RNAi-based response through the up-regulation of *AGO*4 and *RDR*6 in Ma roots.

**Figure 3:**
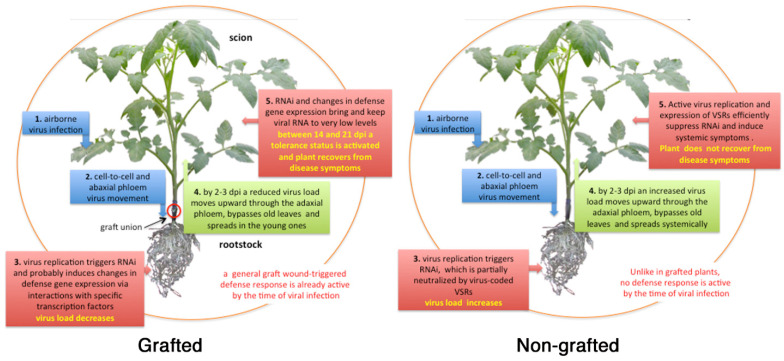
Proposed flow diagram of an airborne virus infection in grafted and non-grafted tomato plants. By the time of virus infection, a general graft wound-response is already active only in grafted plants (pink oval in the background). After cell-to-cell movement in epidermal cells, the virus enters the abaxial phloem, bypasses graft union (circled in red), and accumulates in roots following the source-to-sink phloem sap movement. In rootstock tissues, heavy loads of replicating viral RNA trigger RNAi, probably induces changes in defense gene expression, and signals molecules via interactions with specific transcription factors. Reduced virus loads move from roots into the scion bypassing the graft union and old leaves and spreads into young leaves where it may induce symptoms. Virus load is still sufficient to trigger RNAi and changes in defense gene expression. Between 14-21 days post infection (dpi), virus replication and load are very low, a tolerant state is activated and plant recovers from disease symptoms. In roots of non-grafted plants, the virus replicates actively and accumulates in root tissues by suppressing RNAi via the expression of its VSRs. Then, the virus spreads upward to infect systemically tomato leaves. Active virus replication and expression of its VSRs induce disease symptoms and hinders recovery.

Results with CMV-TTS and CMV-77 in non-grafted plants showed significant differences only in the relative abundance of *DCL*2 transcripts in UC and of *AGO* genes in Ma. On the contrary, viral infection stimulated up-regulation of *DCL*2, *AGO*2, and *RDR*1 in self-grafted UC and of *DCL*2, *AGO*2 and *RDR*6 in the UC/Ma graft combination. Hence, vsiRNAs specific for CMV-TTS and its satRNA were searched in UC leaves in a time-course from 14 to 35 dpi. VsiRNAs derived from the genomic RNA of CMV-TTS were detected only at 14 dpi, i.e., concomitantly with the recovery from diseases symptoms (Table 3). On the contrary, siRNAs against TTS-satRNA were detected at all time points suggesting that host RNAi-triggered degradation of genomic CMV-TTS RNAs was enhanced by its satRNA. This is not surprising, since CMV satRNAs behave as molecular parasites of their helper viruses regardless of whether they attenuate or exacerbate virus-induced symptoms [84]. This conclusion is also compatible with the recovery from disease symptoms shown by Ma and all the graft combination by 30 dpi (Table 3). Estimates of the relative abundance of *DCL*, *AGO,* and *RDR* transcripts were obtained from non-grafted and tomato plants challenged by CMV-77 whereas the early development of necrosis in UC prevented the analysis for this tomato genotype. To partially overcome this limitation, we used the transgenic tomato line UCTC5.9.2 (UCTC) expressing constitutively the Tfn benign variant of CMV satRNAs (Tfn-satRNA), which confers tolerance against CMV-77 [85]. Non-grafted UCTC plants responded to infection of CMV-77 by up-regulation of *DCL*2, *AGO*1, *AGO*2, and *RDR*1 transcripts whereas only *AGO*1 was up-regulated in Ma. On the contrary, a remarkable up-regulation was recorded in all UC/UCTC and Ma/UCTC plants upon infection with CMV-77. RQ values evidenced the up-regulation of *AGO*2 (between 12- and 20-fold), *RDR*1 (between 12- and 20-fold) and *RDR*6 (between 4.5- and 15-fold). *AGO*2 and *RDR*6 showed an up-regulation of 15- and 6.3-fold, respectively, in self-grafted UCTC infected by CMV-77, compared to mock-inoculated controls [63]. A general scheme showing the pattern of RNAi-based response in grafted and non-grafted plants is shown in Figure 3.

### 4.3. Response to Graft Wound and Viral Infection

We used the Ion Torrent RNAseq analysis to identify variations in the transcriptome of grafted and non-grafted Ma and UC tomato plants, exposed or not to the infection of PVY^C^-to. Results provided unique information on gene differentially expressed (DEGs) in the two tomato genotypes in response to graft wound and virus infection. Genes were considered significantly differentially expressed only when log2 of their fold change (FC) expression (log2FC) was ≥1 with a false discovery rate (FDR) ≤ 0.05 [64]. Beyond the use of resistant rootstocks to manage specific soil-borne pathogens of tomato such as fungi, oomycetes, bacteria, and viruses [27,86], it has been suggested that grafting *per se* activates systemic defense mechanisms in response to the graft wound [87,88]. In our study, the graft wound induced 1991 unique DEGs, which accounted for the 5.88% of the total genes (33,810) annotated in tomato genome (Solyc). This percentage was close to the 8% DEGs of the total genes annotated in *A. thaliana* involved in plant wound healing process [89,90]. Most of the graft wound responsive DEGs encoded signal molecules that overlapped their role also in response to abiotic stress and pathogen attack [64,87,90,91]. Table 5 show a selection of such graft wound-responsive genes detected in mock-inoculated and infected tomato plants. Among them APETALA2/ethylene-responsive transcription factor (RAP2/ERF), xyloglucan hydrolase (XTH) and late embryogenesis abundant protein14/light stress-regulated protein3 (LEA14/LSR3) were specifically up-regulated only in Ma/Ma grafted plants. RAP2/ERF are key regulators in various abiotic stress responses, including wounding repair mechanisms and hormone responses such as ethylene and jasmonic acid (JA) [92,93]. Some defense-related genes that are induced by ethylene contain a cis-acting sequence denoted GCC box, which is the core of the ethylene-responsive element and is essential for the expression of several pathogensis-related genes [94]. LEA14 is expressed in response to wound whereas LSR3 is expressed against dehydration that may occur at graft interface before the callus formation at stage 3 of graft union formation [38]. Other genes coding for wound-responsive protein family or maternal effect embryo arrest (MEE) are up-regulated in all graft combinations of mock-inoculated and PVY^C^-to infected plants. MEE14 and MEE59 are involved in blocking the endosperm development [95] and, interestingly, fruits of Ma/Ma and UC/Ma grafted tomato plants did develop very few or no seeds in tests carried out for two consecutive years. The causes leading to this aspermy need a more detailed investigation.

Upon infection of PVY^C^-to, the number of unique DEGs in grafted plants was reduced from 1991 to 1075 with a shift from 5.88% to 3.17% of total Solyc annotated genes, suggesting a diverging contribution of graft wound and viral infection in the number of DEGs. For example, Ma/Ma grafted plants modulated 74.4% and 80% of the unique DEGs in response, respectively, to graft wound and PVY^C^-to. Additionally, the Ma rootstock reduced by 1.5-fold the number of unique DEGs in UC scion compared to self-grafted plants, whereas it did not make any other significant modulation in the transcripts of infected UC/UC and UC/Ma grafted plants. Recent evidences on VSRs coded by PVY^C^-to could account for the decrease in the number of unique DEGs induced by graft wound in grafted plants mock-inoculated. PVY Hc-Pro binds in vivo to 21 nt vsiRNAs during infection in *N. benthamiana* [96] whereas the transgenic expression of Hc-Pro in tobacco altered the expression of hormone-responsive and defense-related genes involved in modifications of cell wall, transcriptional regulation, protein processing, and photosynthesis [97]. In *A. thaliana*, turnip mosaic virus Hc-Pro down-regulated a salicylic acid-mediated response by binding to the salicylic acid (SA)-binding protein SABP3. SABP3 is a tobacco chloroplastic carbonic anhydrase (CA1, SABP3) that was down-regulated in infected UC, Ma, Ma/Ma, and UC/Ma [64].

PVY codes Vpg as an additional VSRs. This protein is involved in the suppression of RNA silencing via the degradation of suppressor of gene silencing 3 (SGS3), which is crucial for the synthesis of dsRNAs derived from viral replication and, in turn, necessary for vsiRNAs production. DCL4/RDR6/SGS3 are thought to be involved in the appearance of the recovery phenotype in virus-infected plants [80]. However, our analysis could not specifically associate a significative up-/down-regulation of *SGS3* with the presence/absence of the recovery.

Thus in this study, the number of graft wound-responsive DEGs were reduced by either the viral infection or the Ma rootstock through mechanisms that will be discussed in the sub-heading 4.4. Most of the DEGs induced by grafting and potyviral infection were involved in the functional categories of signaling/response to stimulus and metabolic processes. PVY^C^-to infection did not exclusively modulated any gene in the photosynthesis functional category. Conversely, 5 genes modulated only in grafted plants (Solyc01g105050.2, Solyc07g054210.2, Solyc07g063600.2, Solyc09g059640.1, Solyc10g007690.2) and 5 genes modulated by graft wound and potyviral infection (Solyc01g105030.2, Solyc06g060310.2, Solyc08g067320.1, Solyc08g067330.1, Solyc09g011080.2) were involved in the photosynthesis functional category and down-regulated.

### 4.4. Oxidative Stress and Antioxidant Enzymes

The production of reactive oxygen species (ROS) is a general event following grafting although it has been proposed that excess of ROS production in grafted cucurbits could be an indication of some graft incompatibility [98,99]. Beyond the implications in graft wound response, rapid production and accumulation of ROS such as O^2−^ and hydrogen peroxide is an important component of the plant response to viral infections both in the infected and non-infected tissues [100]. In infected plants, ROS toxicity can serve as direct protective agent driving the cross-linking of the cell wall or triggering the collapse of challenged host cell at the activation of the hypersensitive response (HR) to generate apoptopic-like signals [101]. In the PVY infection in tomato, the R/*avr* HR may be initiated by the interaction of tomato *pot*-1 resistance gene with the potyviral Vpg [102]. Thus, an up-regulation of genes coding for ROS scavenger enzymes is expected to mitigate ROS toxicity. Indeed, both graft-wounded and infected plants produce antioxidant enzymes such as catalase (CAT), peroxidase (POD) monooxygenase (MO), and manganese or copper/zinc superoxide dismutase (SOD) as ROS scavengers to mitigate ROS toxicity. In compatible grafted plants, the level of such antioxidants is higher than in non-grafted plants, is governed by the rootstock and enables better and strong root development in contrast to low level of antioxidants detected in the incompatible graft interactions [103]. In our study, Solyc03g11780.2 (respiratory burst oxidase homolog D (RbohD)) involved in ROS production was up-regulated in all graft combinations both in mock-inoculated and infected plants, with a seemingly superior up-regulation in mock-inoculated Ma/Ma and in all infected grafted plants (Table 6). Similarly, Solyc11g018800.1 (POD), Solyc12g094620.1 (CAT2) and Solyc04g025650.2 (monooxigenase 1, MO1) were overexpressed in almost all graft combinations both in mock-inoculated and infected plants, whereas Solyc02g082760.2 (CAT2) and Solyc06g049080.2 (MSD1) were significantly down-regulated only in mock-inoculated grafted plants, with the exception of Ma/Ma infected plants (Table 6). These data are in agreement with the notion that in compatible grafted plants, genes coding for ROS scavenger enzymes are up-regulated to compensate for an high ROS production in response to both graft and virus infection.

### 4.5. Resilience and Tolerance

Response to graft wound and viral infection was clearly different in Ma and UC. Our study recorded 91 DEGs in UC plants inoculated with PVY^C^-to and a huge accumulation of viral RNA between 14 and 21 dpi, which probably interfered with recovery from disease symptoms. Conversely, we recorded only 2 DEGs (*DCL2* and *CSD2*) in plants of the Ma ecotype, a reduced accumulation of viral RNA and recovery from disease symptom upon infection of the three taxonomically distinct viruses used in our studies. Thus, Ma was refractory to changes in gene expression and resilient to disease symptoms. *DCL2* and *CSD2* were up-regulated in Ma, suggesting that it employed few resources in response to virus infection but specific enough to recover from disease symptoms. According to Kørner et al. [80], recovered tissues may be in a “tolerant state,” characterized by reduced levels of viral RNA and accumulation of vsiRNAs. Maintenance of a virus infection in recovered leaves has a beneficial impact also on the resilience of plants to abiotic stresses and drought [104]; a characteristic that we did not test. Conversely, the up-regulation of resistance genes requires energy resources, which are generally recruited from primary metabolism and plant growth and development [105]. For example, leaves are generally source tissues but upon infection of airborne pathogens, such as viruses, they strengthen cell wall and down-regulate genes involved in photosynthesis [105]. In UC, we detected 18 down-regulated genes involved in photosynthesis in response to both PVY^C^-to infection and graft wound. Therefore, we propose that the Ma response to the infection of TSWV, CMV, and PVY could be a case of tolerance, which is now reconsidered as “a mitigation of the impact of virus infection irrespective of the pathogen load” [104,105,106,107].

## 5. Conclusions

Severe strains of TSWV, CMV, and PVY are arthropod-borne pathogens that induce devastating crop diseases worldwide. Current control methods are unsustainable whereas grafting offers a promising environmentally friendly alternative. The results of our studies suggest that grafting may represent a rapid and sustainable approach to deal quickly and mitigate the negative effects of airborne viral infections in tomatoes when useful resistance genes are not available or have not yet been identified. The Manduria tomato ecotype has proven to be an adaptive rootstock, considering its tolerance to high virus loads and resilience to the appearance of symptoms induced by three taxonomically distinct arthropod-borne viruses. However, the graft wound itself activates defense responses that impact infection as demonstrated by susceptible varieties which, when grafted on themselves, showed levels of tolerance to viral infection and resilience to the appearance of symptoms very similar to those found in the Manduria ecotype. Thus grafted plants probably operate with higher energy resources compared to non-grafted counterparts and necessary to respond to wounding. At the same time, the energy resources contribute to prevent over-accumulation and expression of viral RNAs. This equilibrium limits the cost for the host while saving resources by reducing the number of DEGs necessary to respond to viral infection.

In principle, the adoption of vegetable grafting in agriculture requires an accurate selection of the rootstock to cope with specific biotic and abiotic stresses. Collectively our studies offer an unprecedented view of grafting based on tolerance rather than the resistance that might be fruitfully applied to a wide range of pathogens such as fungi, oomycetes, and bacteria, as well as other virus species.

## Figures and Tables

**Table 1:** Virus species recorded in Italian tomato crops.

Virus	Acronim	Transmission
Alfalfa mosaic virus	AMV	aphids
Cucumber mosaic virus	CMV	aphids
Parietaria mottle virus	PMoV	thrips
Pelargonium zonate spot virus	PZSV	pollen, thrips
Pepino mosaic virus	PepMoV	contact
Potato virus Y	ToMV	aphids
Tobacco mosaic virus	TMV	contact
Tomato mosaic virus	ToMV	contact/aerosol
Tomato brown fruit rugose virus	ToBFRV	contact
Tomato cholorosis virus	ToCV	aleyrodids
Tomato infectious chlorosis virus	TICV	aleyrodids
Tomato spotted wilt virus	TSWV	thrips
Tomato yellow leaf curl virus	TYLCV	aleyrodids
Tomato yellow leaf curl Sardinia virus	TYLCSV	aleyrodis

**Table 2:** Response of *Solanum* spp. genotypes, tomato varieties, and graft combinations to challenge inoculation of Sw5-RB TSWV isolate CiPz.

	14 *	21 *	21 *	28 *
**Non-Grafted Plants**	**Symptoms**	**Symptoms**	Viral RNA Load **	**Symptoms**
UC82 (UC)	VN, LE	LY	2.63 ± 0.08 ^a^	PD
Manduria (Ma)	mLD	R	0.85 ± 0.02 ^e^	R
Regina (Re)	VN, LE	LY	1.85 ± 0.01 ^c^	PD
Sw5-Messapico (Me)	VN, LE	LY	1.91 ± 0.01 ^c^	PD
Sw5-Faino (Fa)	VN, LE	LY	2.0 ± 0.03 ^bc^	PD
Pullrex (Pu)	LY	D	2.1 ± 0.04 ^b^	PD
Molfettese (Mo)	mMos	R	0.78 ± 0.02 ^e^	R
*Solanum integrifolium*	mMos	R	0.87 ± 0.03 ^e^	R
*S. nigrum*	sMos, sLD	N, sLD, LE	1.9 ± 0.09 ^c^	N
*S. torvum*	mMos	mMos	1.25 ± 0.06 ^d^	mMos
Grafted Plants ***	**Symptoms**	**Symptoms**	Viral RNA Load **	**Symptoms**
UC/Mo	Mos	R	0.19 ± 0.02 ^c,d,e^	R
UC/*S. integrifolium*	sMos, LE	sLD, LY	0.35 ± 0.06 ^a^	N
UC/Ma	Mos	R	0.10 ± 0.06 ^e,f,g^	R
UC/UC	mMos	R	0.04 ± 0.008 ^f,g^	R
Ma/Ma	mMos	R	0.01 ± 0.003 ^g^	R
Me/Mo	sMos,	sMos, LY	0.30 ± 0.07 ^a,b,c,d^	LY, sLD
Me/*S. integrifolium*	sMos,	sMos, LY	0.31 ± 0.07 ^a,b,c^	LY, sLD
Me/Ma	mMos	R	0.01 ± 0.004 ^g^	R
Me/Me	mMos	R	0.02 ± 0.006 ^g^	R
Fa/Mo	Mos	Mos	0.22 ± 0.04 ^b,c,d,e^	R
Fa/*S. integrifolium*	LE, VN	LY, VN	0.38 ± 0.04 ^a^	N
Fa/Ma	mMos	R	0.12 ± 0.04 ^e,f,g^	R
Fa/Fa	mMos	R	0.12 ± 0.06 ^e,f,g^	R
Pu/Mo	sMos	sLD, VN	0.34 ± 0.02 ^a,b^	N
Pu/*S. integrifolium*	LE, VN	LY, VN	0.36 ± 0.03 ^a^	SLD, VN
Pu/Ma	Mos	LE, VN	0.18 ± 0.04 ^d,e^	LY, VN
Pu/Pu	Mos	R	0.16 ± 0.03 ^e,f^	R

* = days post-inoculation; ** = estimates of accumulation of viral RNA by quantitative dot blot hybridization at 21 dpi. Chemiluminescent hybridization signals were used to detect and quantify the accumulation of viral RNA by using *Glyceraldehyde 3-phosphate dehydrogenase* (*GAPDH*) as housekeeping gene for normalization. Each value represents average of three biological replicates ± standard error among replicates. Letters represent statistically significant differences values according to ANOVA analysis for non-grafted and grafted plants using Tukey’s test (*p* ≤ 0.05); *** = scion/rootstock; LE = leaf epinasty; LY = leaf yellowing; mLD = mild leaf distortion; mMos = mild mosaic; Mos = mosaic; N = whole leaf necrosis; PD = plant death; R = Recovery; sLD = severe leaf distortion; sMos = severe mosaic; VN = vein necrosis. Sw5 = plant expressing Sw5 resistance gene.

**Table 3:** Response of tomato varieties UC82 (UC) and Manduria (Ma) and their graft combinations to infection of CMV-TTS and CMV-77.

CMV-TTS *	15 ***	21 ***	21 ***	30 ***
**Plants**	**Symptoms**	**Symptoms**	**Viral RNA Load ^†^**	**Symptoms**
UC	TS, LD	sTS	0.16 ± 0.008 ^b^	sTS
Ma	TS, LD	R	0.25 ± 0.001 ^a,b^	R
UC/Ma ^§^	TS	R	0.33 ± 0.021 ^a^	R
UC/UC ^§^	TS	R	0.31 ± 0.025 ^a^	R
Ma/Ma ^§^	TS	R	0.3 ± 0.007 ^a^	R
CMV-77 **	15 ***	15 ***	21 ***	30 ***
**Plants**	**Symptoms**	**Viral RNA Load ^†^**	**Symptoms**	**Symptoms**
UC	SN, LN	0.04 ± 0.001 ^c^	PD	PD
Ma	mS	0.33 ± 0.025 ^a,b^	tR	PD
UC/Ma ^§^	SN, LN	0.24 ± 0.07 ^b^	SN, LN ^¥^	SN, LN
UC/UC ^§^	SN, LN	0.43 ± 0.06 ^a^	SN, LN	SN, LN
Ma/Ma ^§^	YBL	0.03 ± 0.001 ^c^	R	R

* = supporting TTS satRNA; ** = supporting 77 satRNA; *** = days post-inoculation (dpi); ^†^ = ng of viral RNA estimated by quantitative dot blot hybridization at 15 and 21 dpi. Chemiluminescent hybridization signals were used to detect and quantify the accumulation of viral RNA by using *GAPDH* as housekeeping gene for normalization. Each value represents average of three biological replicates ± standard error among replicates. Letters represent statistically significant differences values according to ANOVA analysis for non-grafted and grafted plants using Tukey’s test (*p* ≤ 0.05); ^§^ = scion/rootstock; LD = leaf distortion; LN = leaf necrosis; mS = moderate stunting; PD = plant death; SN = stem necrosis; TS = top stunting; sTS = severe top stunting; R = recovery; tR = transitory recovery; YBL = yellowing of basal leaves; ^¥^ = necrosis developed in 1 out of 4 challenged plants.

**Table 4:** Response of tomato varieties UC82 (UC) and Manduria (Ma) and their graft combinations to the infection of PVY^C^-to.

	14 *	14 *	21 *	21 *
Plants	Symptoms	Viral RNA Load ***	Symptoms	Viral RNA Load ***
UC	Mos, LD	5.72 ± 1.34 ^b^	sMos, sLD	16.07 ± 1.96 ^a^
Ma	mLD	8.03 ± 0.93 ^b^	R	2.22 ± 0.68 ^c^
UC/Ma **	mS	17.92 ± 1.84 ^a^	R	4.69 ± 1.16 ^bc^
UC/UC **	A	13.68 ± 2.4 ^a^	A	6.03 ± 0.44 ^b^
Ma/Ma **	mLD	16.07 ± 1.96 ^a^	R	2.39 ± 1.30 ^c^

* = days post inoculation (dpi); ** = scion/rootstock; *** = pg of viral RNA estimated by quantitative dot blot hybridization at 14 and 21 dpi. Chemiluminescent hybridization signals were used to detect and quantify the accumulation of viral RNA by using *GAPDH* as housekeeping gene for normalization. Each value represents average of three biological replicates ± standard error among replicates. Letters represent statistically significant differences values according to ANOVA analysis for non-grafted and grafted plants using Tukey’s test (*p* ≤ 0.05); Mos = mosaic LD = leaf distortion; mLD = moderate leaf distortion; mS = moderate stunting; sMos = severe mosaic; sLD = severe leaf distortion; R recovery; A = asymptomatic.

**Table 5:** Significative differential expression of graft-responsive genes in mock-inoculated and infected tomato plants.

	Mock			Infected		
Locus Name	Ma/Ma *	UC/UC *	UC/Ma *	Ma/Ma *	UC/UC *	UC/Ma *
Solyc11g072600.1 (RAP2/ERF4) ***	3.20 **			3.63		
Solyc04g080700.2 (wound-responsive family protein)	3.57	3.26	2.55	2.51	2.86	2.61
Solyc07g056000.2 (XTH)			1.74		2.55	
Solyc07g052980.2 (XTH)	1.27			2.24		
Solyc02g091920.2 (XTH)	−1.58	−1.94	−1.61	−1.44		
Solyc09g075210.2 (AtLEA5/SAGS21)	−3.29	−4.33	−3.41	−4.52	−1.93	−2.13
Solyc06g006000.2 (MEE59)	5.03	5.51	5.49	3.75	4.23	4.25
Solyc01g108910.2 (MEE14)	5.41	5.03	5.00	5.00	4.54	4.64
Solyc01g095140.2 (LEA14, LSR3)	2.30			3.42	2.43	2.21

* = Scion/rootstock graft combination; ** = significantly differentially expressed genes (DEGs) with *p* value adjusted for multiple testing with the Benjamini–Hochberg procedure which controls false discovery rate (FDR) ≤ 0.05; *** = Arabi name in parentheses; RAP5/ERF4 = APETALA5/ethylene-responsive transcription factor 4; XTH = xyloglucan endotransglucosilase/hydrolase; AtLEA5/SAGS21 = late embryogenesis abundant protein 5/senescence-associated gene 21; MEE14,MEE59 = maternal effect embryo arrest; LEA14/LSR3 = late embryogenesis abundant protein/light stress protein.

**Table 6:** Significative differential expression of graft-responsive antioxidant genes in mock-inoculated and infected tomato plants.

Locus Name	GO *	Mock			Infected		
		Ma/Ma **	UC/UC	UC/Ma	Ma/Ma	UC/UC	UC/Ma
**Genes Involved in ROS Production**							
Solyc03g117980.2 (RbohD) ***	GO:0004601	2.79 ^†^	1.51	1.56	2.97	2.17	2.21
**Genes Involved in ROS Scavenging**							
Solyc01g006300.2 (POD2)	GO:0004601 GO:0006979 GO:0042744	3.38	2.45	2.54			
Solyc11g018800.1 (POD)	GO:0004601 GO:0006979 GO:0042744	4.21	3.10	2.89	4.05	4.04	4.18
Solyc02g082760.2 (CAT2)	GO:0004096 GO:0006979 GO:0042744	−1.76	−2.05	−2.22	−1.69		
Solyc12g094620.1 (CAT2)	GO:0004096 GO:0042542 GO:0042744	3.88	2.59	2.05		1.95	2.12
Solyc04g025650.2 (MO1)	GO:0004497	1.69	2.13	2.07	3.32	3.22	3.12
Solyc06g049080.2 (MSD1)	GO:0004784	−1.51	−1.48	−1.49			

* = Gene ontology involved in ROS response; ** = Scion/rootstock graft combination compared to non-grafted plants; *** = Arabi name in parentheses; RbohD = Respiratory burst oxidase homologue D; POD = Peroxidase 2; CAT = Catalase; MO = Monooxygenase; MSD = Manganese superoxide dismutase; ^†^ = significantly differentially expressed genes (DEGs) with *p* value adjusted for multiple testing with the Benjamini–Hochberg procedure which controls false discovery rate (FDR) ≤ 0.05.
